# Dietary and Lifestyle Patterns in the Spanish Pediatric Population (One to <10 Years Old): Design, Protocol, and Methodology of the EsNuPI Study †

**DOI:** 10.3390/nu11123050

**Published:** 2019-12-13

**Authors:** Casandra Madrigal, María José Soto-Méndez, Ángela Hernández-Ruiz, Emma Ruiz, Teresa Valero, José Manuel Ávila, Federico Lara-Villoslada, Rosaura Leis, Emilio Martínez de Victoria, Jose Manuel Moreno, Rosa M Ortega, María Dolores Ruiz-López, Gregorio Varela-Moreiras, Ángel Gil

**Affiliations:** 1 Department of Nutrition and Food Sciences, Faculty of Pharmacy, University of Granada, 18071 Granada, Spain; casandram@correo.ugr.es; 2 Iberoamerican Nutrition Foundation (FINUT), Armilla, 18016 Granada, Spain; msoto@finut.org (M.J.S.-M.); ahernandez@finut.org (Á.H.-R.); agil@ugr.es (Á.G.); 3 CIBERESP, Consortium for Biomedical Research in Epidemiology and Public Health, Carlos III Health Institute, 28029 Madrid, Spain; e.ruiz@externos.isciii.es; 4 National Center for Epidemiology, Carlos III Health Institute, 28029 Madrid, Spain; 5 Spanish Nutrition Foundation (FEN), 28010 Madrid, Spain; tvalero@fen.org.es (T.V.); jmavila@fen.org.es (J.M.Á.); gvarela@ceu.es (G.V.-M.); 6 Instituto de Nutrición Puleva, 18004 Granada, Spain; federico.lara@lactalis.es; 7 Department of Pediatrics, Unit of Pediatric Gastroenterology, Hepatology and Nutrition, University Clinical Hospital of Santiago, 15705 Santiago de Compostela, Spain; mariarosaura.leis@usc.es; 8 CIBEROBN (Physiopathology of Obesity and Nutrition CB12/03/30038), Instituto de Salud Carlos III (ISCIII), 28029 Madrid, Spain; 9 Department of Physiology, University of Granada, 18071 Granada, Spain; emiliom@ugr.es; 10 Institute of Nutrition and Food Technology ”José Mataix”, Biomedical Research Center, University of Granada, 18100 Granada, Spain; 11 Department of Pediatrics, University of Navarra Clinic, 28027 Madrid, Spain; jmorenov@unav.es; 12 Department of Nutrition and Food Sciences, Faculty of Pharmacy, Complutense University, 28040 Madrid, Spain; rortega@ucm.es; 13 Department of Pharmaceutical and Health Sciences, Faculty of Pharmacy, CEU San Pablo University, 28668 Madrid, Spain; 14 Department of Biochemistry and Molecular Biology II University of Granada, University of Granada, 18071 Granada, Spain

**Keywords:** dietary habits, feeding behaviors, food intake, physical activity, sedentary behaviors, pediatrics, Spanish children, dairy products, infant formula, EsNuPI study

## Abstract

The interest in a healthy diet and lifestyle during the early stages of life increased, pointing out its role in the development of noncommunicable chronic diseases throughout adult life. Dietary habits and dietary patterns begin to be established in early childhood and persist during adulthood. Therefore, the EsNuPI (“Nutritional Study in Spanish Pediatric Population”) study aims to depict the dietary patterns, physical activity, and sedentary behaviors in Spanish children aged from one to <10 years old. This prospective, cross-sectional, observational study recruited a total of 1514 children from Spanish cities with >50,000 inhabitants, stratified by Nielsen areas. Participants were involved in one face-to-face survey, followed by a telephone survey after at least one week. Information about dietary intake and habits was obtained using a quantitative food frequency questionnaire and two 24-h dietary recalls. Physical activity and sedentary behaviors were registered using a specific questionnaire based on a seven-day record. Data were processed and stratified by categorical variables to be statistically analyzed in order to meet the study objectives. This study is the first of its kind in a Spanish reference population of this age range and the first to evaluate whether the consumption of adapted milk formulas and dairy products is associated with healthier dietary patterns and better diet quality and lifestyles in this group.

## 1. Introduction

The interest in a healthy diet and lifestyle during the early stages of a child’s life increased, pointing out its role in the development of noncommunicable chronic diseases (NCCDs) throughout life [1]. At later ages, in addition to favoring optimal growth and development, feeding constitutes a means for the acquisition of healthy eating habits, which have an impact on nutritional behavior in the short, medium, and long term. Dietary habits and dietary patterns begin to be established in early childhood, are consolidated before the end of the first decade of life and persist during adulthood [2].

Technological advances and environmental and social changes that took place in recent years also changed lifestyles and dietary patterns, conditioning the prevalence of overweight, obesity, and NCCDs such as hypertension, type 2 diabetes, and cardiovascular disease. Currently, there are only a few grouped data on the dietary habits of the European pediatric population [3,4]. The World Health Organization (WHO) publishes data in relation to healthy habits in children aged 11–15 years, but not in younger children [5].

A systematic analysis for the Global Burden of Disease found that, in 2017, 11 million deaths and 255 million disability-adjusted life-years were attributable to dietary risk factors. High intake of sodium, low intake of whole grains, and low intake of fruits were the leading dietary risk factors for death and disability-adjusted life-years globally and in many individual countries [6].

In Spain, very few studies were conducted on dietary habits and lifestyle behavior in the pediatric population. The two most important were the ALSALMA (from the Spanish “Alimentando la salud del mañana”) [7] and the ENALIA (“National Dietary Survey on the Child and Adolescent Population in Spain”) studies [8], both of them cross-sectional studies, carried out in 2013 and 2013–2014, respectively.

The ALSALMA study was conducted in a representative sample of Spanish children 0–36 months (*n* = 1559, 54% boys), and the ENALIA study was carried out on a national sample of children and adolescents aged from six months to 17 years (*n* = 1862, 50% girls) [7,8].

Finally, in 2015, the ANIBES (“Anthropometry, Intake and Energy Balance”) study was published; ANIBES was a cross-sectional study performed in the Spanish population, including children and adolescents between nine and 17 years old (*n* = 2009, 50.4% males) [9].

Also, European studies that involve the Spanish population were carried out, e.g., the IDEFICS (“Identification and prevention of dietary- and lifestyle-induced health effects in children and infants”) study that included a cohort study (I.Family) which is a population-based multi-center study of children aged 2–9 years recruited in selected and specific regions of eight European countries (including only the cities of Zaragoza and Huesca in Spain). These two studies published several reports describing dietary intake, dietary patterns, and the changes in children’s diet over time [10,11,12].

In addition, a review of the evidence was conducted to evaluate associations between milk or dairy product intake and health outcomes in children and adolescents (2–19 years). Available data indicated that childhood dairy product consumption may affect various facets of growth and development, and evidence suggests that dairy products are important for linear growth and bone health [13,14].

Moreover, Santaliestra-Pasías et al. studied the relationship between specific lifestyle behaviors and dairy consumption in a sample of European children and adolescents from the IDEFICS study. The study found that European children with a healthy lifestyle, specifically regarding physical activity and sedentary behaviors over time, consumed more milk and yogurt. This study suggests that the protective effect of dairy products on cardiovascular diseases (CVD) could be related to the association between their consumption and specific lifestyle behaviors [15].

In Spain, Ortega et al. studied the association between dairy consumption and dietary patterns and intake of selected nutrients in 7–11-year-old children and concluded that children who drank more milk also had better dietary patterns [16].

Unfortunately, no precise data for adapted and fortified milk formulas (those whose composition is adapted to the nutritional needs of the child population) are available in Spain. However, they are consumed at around 9225 tons per year and are gaining importance and increased sales [17]. According to the Food Consumption Report in Spain 2018, sales of fortified milk increased by 26.3% compared to 2017 [18].

As already stated, there is a paucity of information about dietary patterns, dietary habits, and physical activity and sedentary behaviors in Spanish children in the one- to <10-year-old age group, and there is also no information about any possible association between these patterns, habits, and behaviors and the consumption of adapted and fortified milk formulas and dairy products. Therefore, the EsNuPI (“Nutritional Study in Spanish Pediatric Population”) study is aimed at depicting the food consumption, nutrient intake, and dietary patterns, as well as physical activity and sedentary behaviors of Spanish children from one to <10 years old in urban areas with >500,000 inhabitants, distributed across nine geographical areas (according to the Nielsen Spanish criteria), and it is aimed at evaluating whether or not the regular consumption of dairy products and adapted and fortified milk formulas is associated with the quality of the diet. 

To that end, the specific objectives of the EsNuPI study were (1) to characterize the dietary energy, food consumption, and nutrient intake, as well as the dietary patterns and habits of the study population, (2) to evaluate the dietary quality through the determination of dietary diversity and variety, as well as through the use of diverse dietary quality indexes, (3) to describe the physical activity and sedentary behaviors and to evaluate them according to the existing activity and resting recommendations for this age group, and according to the World Health Organization’s growth standards, (4) to combine data on diet and physical activity in order to determine the over- and under-reporters of energy consumption, (5) to stratify the population according to different categorical variables (e.g., sex, age, socioeconomic status, household habits, etc.), and test for any possible differences or interactions in dietary patterns and diet quality, and (6) to compare all different variables of the study among the urban Spanish reference population and the booster sample of adapted and fortified milk formula consumers.

## 2. Experimental Section

### 2.1. Pilot Study

Even though the final fieldwork was carried out from October 2018 to January 2019, a pilot study was previously carried out in September 2018 by MADISON MK, the company in charge of data collection and processing for the EsNuPI study. For this purpose, 25 interviews were conducted in several geographical locations across Spain. Once the results from the pilot study were completed, the limitations were detected and corrected. Then, the questionnaires were approved for the final protocol.

### 2.2. Study Design

The EsNuPI study is a prospective, cross-sectional, observational study. The EsNuPI study aimed to have a sample size representative of children living in Spain (excluding the autonomous cities of Melilla and Ceuta) aged one to <10 years and living in urban areas with >50,000 inhabitants. These urban areas represent 1.8% of the total number of municipalities of Spain and 52.6% of the total Spanish children population of one to <10 years old [19].

The sample of the EsNuPI study was calculated based on the 2018 census data published by the Spanish Bureau of Statistics (“Instituto Nacional de Estadistica” (INE)) [19] and stratified appropriately by age, sex, Nielsen geographical area, population size, and type of milk consumed.

### 2.3. Inclusion and Exclusion Criteria 

Inclusion and exclusion criteria involved the ability to eat a diversity and variety of food items; Table 1 details all these criteria.

### 2.4. Sample

The sample was randomized in multiple stages and stratified according to sex, age, population size, and Spanish Nielsen areas. Two subsamples were selected for the EsNuPI Study; one subsample is representative of the urban, non-vegan Spanish population from one to <10 years old, and the other subsample is a booster of non-vegan one to <10-year-old Spanish children who lived in urban areas and regularly consumed adapted and fortified milk formulas. The initially estimated sample was 1500 individuals, and the sample errors were ±2.52% and ±2.59%, respectively, for a 95.5% confidence level and estimation of equally probable categories (*p* = q = 50%), considering a universe of 2,205,646 children.

For the final sample, 1514 parents or caregivers signed consent forms and completed the first interview (face-to-face) providing sociodemographic information, answering a quantitative food frequency questionnaire (FFQ), a physical activity and sedentary behaviors questionnaire (PABQ), and the first 24-h dietary recall (24-h DR). Later, only 1448 subjects completed the study by answering the telephone survey that collected the second 24-h DR. Details of the use of these questionnaires are described below.

The final sample consisted of two subsamples, children representative of the urban Spanish population (vegans excluded), hereinafter called the “reference population”, and a “booster” sample that consumed adapted and fortified milk formulas. For this study, the following were considered as milk formulas: infant formula, follow-on milk formula, toddler’s milk formula (also termed in Spain “growing up” milk formula), and fortified milk formula (e.g., docosahexaenoic acid (DHA), calcium, vitamin D, iron).

**Figure 1:**
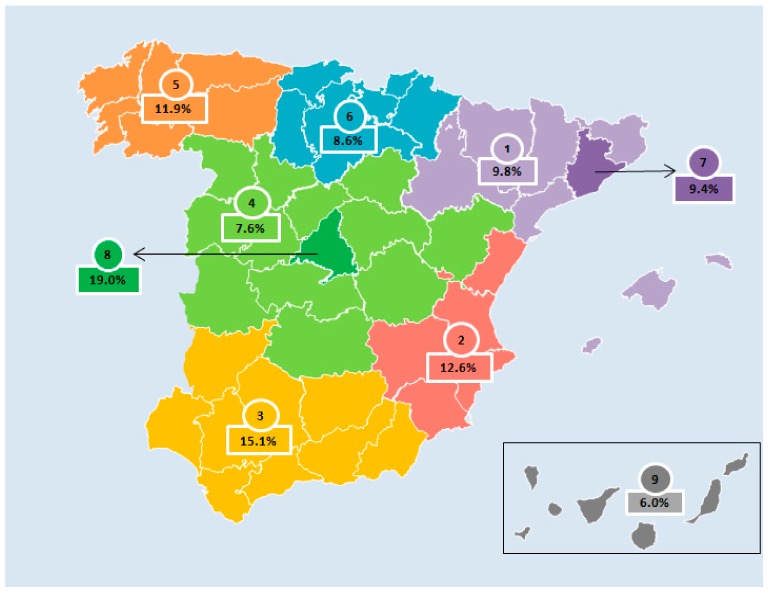
Nielsen geographical distribution of the sample for the EsNuPI study: Area 1 (9.8%), northeast; Area 2 (12.6%), Levant (east); Area 3 (15.1%), south; Area 4 (7.6%), central; Area 5 (11.9%), northwest; Area 6 (8.6%), north central; Area 7 (9.4%), metropolitan area of Barcelona; Area 8 (19.0%), metropolitan area of Madrid; Area 9 (6.0%), Canary Islands.

In addition, considering the need for all age and sex groups to be represented, the sample was stratified (50% boys, 50% girls, one to <3 years old, three to <6 years old, and six to <10 years old). Table 2 shows the distribution of the targeted and final samples, and Figure 1 shows the distribution of the sample by Nielsen areas.

Individual quotas were defined for all the variables, which allowed the identification of the total number of interviews required to properly represent the sociodemographic distribution under study. The sample design and the high number of sampling points determined the number of required interviewers and their interviewing area. 

### 2.5. Ethical Aspects 

The final protocol followed the ethical standards recognized by the Declaration of Helsinki of 1964 and was approved by the Research Ethics Committee of the University of Granada (Spain), coded as “659/CEIH/2018” in September 2018. Thereafter, the study was registered at ClinicalTrials.gov (Protocol Registration and Results System—PRS—Receipt Release Date: 22 April 2019; Unique Protocol ID: FF01/2019).

Before any information request, during the interview, the study was explained to the parents or legal guardians. Children whose parents or caregivers did not provide a signed consent form or who did not complete any study phase were excluded from the study.

Participants of the study could stop the interviews at any moment, and their collected partial information was excluded from any analysis.

#### Participant Confidentiality

MADISON MK (TELECYL. S.A.) was responsible for collecting all personal data of respondents in order to manage and carry out the survey. MADISON MK is knowledgeable and adapted to the new General Data Protection Regulation of the European Union (EU) 2016/679. Each participant gave their express consent to participate in the survey. Once the data collection and the survey verification work were finished, the files obtained were anonymized for their processing.

### 2.6. Survey Instruments

All the survey instruments were adapted for the study and were designed, verified, and modified previously in the pilot study. Below, a brief description of the survey instruments that were used for this study is given.

#### 2.6.1. Sociodemographic and Personal Questionnaire

This questionnaire allowed the researcher to know the characteristics of the study population. The questionnaire included date and place of birth, nationality, gender, educational level of the parent or caregiver (elementary or less/secondary/university/higher education), place of residence (Nielsen area), employment situation (unemployed/employed), household information (household members/family feeding/lifestyle factors), professional occupation (administrative/farmers/professionals), working times (working days/working hours), and household income. Moreover, the questionnaire was used to determine if participants met the inclusion criteria.

#### 2.6.2. Food Frequency Questionnaire

Dietary diversity and variety, as well as an estimation of food consumption, were measured using a quantitative FFQ. For our study, an FFQ was used that was previously modified, adapted and validated with the portion sizes and food groups that the Spanish child population usually consumes [20,21]. This FFQ was a quantitative one, including 10 food groups and 160 items, and with foods and beverages most commonly consumed by the Spanish population, with standard portion sizes for each item using the common units of weight (g) and of volume (mL) [22]. These groups were divided into subgroups due to the great diversity of food products that currently exists. The main food groups and subgroups, and the specific portion sizes that were considered in this study are shown in Table 3.

Participants were asked to answer questions regarding their child’s usual food consumption over the last 12 months and indicate how frequently they consumed these foods from the options of never, rarely, monthly, weekly, and daily.

#### 2.6.3. Twenty-Four-Hour Dietary Recall

Food consumption can be estimated using different methods, and the most common for quantitative assessment of the intake of energy and nutrients from the data obtained is the 24-h DR [23,24]. For this study, an ad hoc questionnaire was designed and previously verified and modified during the already explained pilot study. 

For the 24-h DR, the participants or their caregivers recalled all food and beverage intake at home and away from home in the last 24 h (one day’s intake). For this study, two non-consecutive recalls, including weekdays and weekend days, were developed; the first one was performed through a face-to-face interview, and the second one was performed through a telephone call. For the 24-h DR, we followed the guidance on the EU menu methodology of the European Food Safety Authority [25].

The information obtained was structured as mealtimes (breakfast, mid-morning, lunch, mid-afternoon, dinner, and other moments). Subjects were also asked about the place (home, school, in another house, outside in a restaurant) and time of meal consumption.

The participant made a detailed description of the food consumption (ingredients, method of preparation, and brands), and this information allowed the correct coding and weight assignment for each food item. To facilitate the process, the interviewers used the “tables of common home measures and habitual portion sizes for Spain population” [26] and the “photo guide of common portions sizes of Spanish foods” [27]. The latter photo guide includes 12 food groups, 204 foods commonly consumed in the Spanish population, and 944 photographs.

#### 2.6.4. Physical Activity and Sedentary Behavior Questionnaire

Questionnaires for the assessment of physical activity were indicated as useful instruments because they are easy to manage, with a low cost, and they can extract information from numerous samples in a short interval of time [28].

The PABQ used for this study was a modification of a questionnaire previously validated in children <10 years from Colombia based on a seven-day record [29]. Small modifications, mostly in commonly used language terms, were made to this questionnaire to adapt it to the needs of the present study.

Physical activity was reported indicating all the activities performed by the child in one day (24 h) during the last week (a seven-day record), including hours of sleep and screen time, and was reported separately for weekdays and weekends.

This questionnaire included activities that require more effort (cycling, walking, dancing, jumping, etc.) and activities that require little or no effort (reading time/homework, hours spent watching television, time spent using the computer and game consoles or screens, foreign language classes, music or drawing courses). Also, participants could fill in other activities that were not included in the other sections.

Finally, sleeping and eating habits included the number of hours each child slept per night and duration of naps, as well as the number of hours eating, on average, and they were reported separately for weekdays and weekends.

### 2.7. Stages of the Fieldwork

For the fieldwork process, the following two stages were achieved:

1. Personal survey: the initial contact with the interviewer and the face-to-face interview.
2. Telephone survey: contact with the interviewer through a telephone call.

Both surveys were carried out with the children’s parents or caregivers, and, when it was possible, in the presence of the child to try to complete the information.

#### 2.7.1. Stage 1, Personal Survey: Initial Contact with the Interviewer and Face-To-Face Interview

The initial contact with the possible participants was made by different approaches, e.g., outside schools, daycare centers, playgrounds, and recreational parks. The interviewer verified through a filter questionnaire that the participant was eligible for the EsNuPI study according to the abovementioned inclusion and exclusion criteria; if criteria were met, the interviewer set an appointment with the parent or caregiver.

The face-to-face interviews were always conducted at home, and there was only one interview per subject of each house. The computer application CAPI (Computer-Assisted Personal Interviewing) was used for the collection of the data according to the guidance of the EU menu methodology [25] in order to make this process easier and more efficient.

The first interview (face-to-face) with an approximate duration of 120 min comprised the following items:

1. Classification and sociodemographic questionnaire (18 min).
2. Anthropometric data (2 min): weight and size data declared by parents or caregivers based on the child’s health card.
3. FFQ (60–90 min).
4. 24-h DR (20 min).
5. PABQ (15 min).

No prior notification was given to the subjects about whether or when they would be interviewed about their food consumption.

#### 2.7.2. Stage 2, Telephone Survey: Contact with the Interviewee through a Telephone Call

The data collection was done by telephone using the CATI (Computer-Assisted Telephone Interviewing) computer application by the same previously trained interviewers [25].

The second interview (“telephone survey”) with an approximate duration of 20 min comprised the following item:

- Second 24-h DR, ≥7 days after the first survey, considering that one should collect information about weekdays and the other about weekends.

Once stages 1 and 2 were completed and the quality of the information was assured, the verification of all data provided by the interviewers was carried out. This verification was done by MADISON MK at the end of each working day. In those cases where it was necessary to complete information by the participants, MADISON MK proceeded to contact them and verify the missing data. 

**Figure 2:**
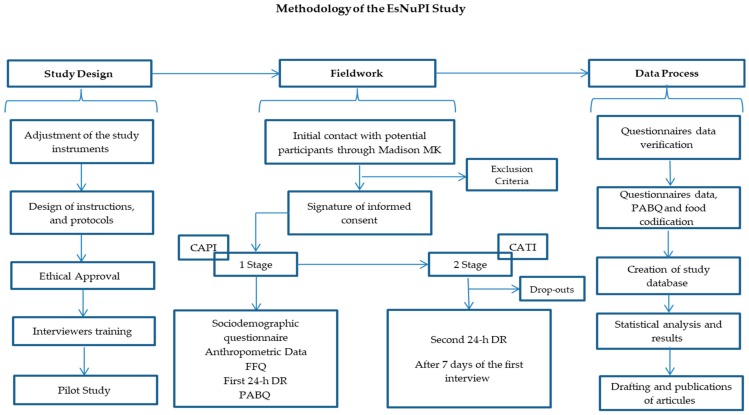
Design and methodology of the EsNuPI study. CAPI, Computer-Assisted Personal Interviewing; FFQ, food frequency questionnaire; 24-h DR, 24-h dietary recall; PABQ, physical activity and sedentary behaviors questionnaire; CATI, Computer-Assisted Telephone Interviewing.

Subsequently, the information submitted was verified and the suitable corrections were applied and approved by the dieticians/nutritionists from the FEN (“Spanish Nutrition Foundation”) and FINUT (“Iberoamerican Nutrition Foundation”). Finally, the information was sent back to MADISON MK, who considered all the changes proposed to create the final EsNuPI database. Figure 2 shows the scheme for the methodological process of the EsNuPI study.

### 2.8. Quality Control

The quality control of the collected information was supervised by trained personnel, according to the following protocol:

- Necessity to answer all the questionnaires’ items.
- The initial quality control was based on the descriptions sent by the participants and combined with the photo guide information. Special attention was given to validate some variables such as ingredients, brands of processed and fast food, portion size, or culinary technique in order to obtain adequate information for future codification.
- FEN and FINUT were responsible for checking the food consumption records during the study.
- From the beginning of the coding process, FEN and FINUT worked together with MADISON MK to check the information and give them individual feedback on their work.
- The surveys received throughout the fieldwork were coded and depurated, transferring continuous feedback to the interviewers about possible errors or inconsistencies in their completion.
- Verification calls were performed by each interviewer to verify the incomplete answers and correct possible inconsistencies.
- The questionnaires that did not pass the stipulated quality controls were removed.
- The final approval of the received information was given by FEN and FINUT.

### 2.9. Statistical Analysis 

A database was created with the information that was obtained from the two fieldwork stages. All data were coded, depurated, and processed to generate a database with all the variables for their statistical treatment, using the software IBM SPSS Statistics for Windows, Version 20.0 (IBM Corp., Armonk, NY, USA) and R Studio 3.5.1 for Windows (R Core Team, Vienna, Austria).

A descriptive analysis of the analyzed variables was performed according to the distribution of each variable, and results are expressed as means ± standard deviation (SD), median, ranges, percentiles, and percentages for numerical variables, and in absolute and relative frequencies (*n* and %) for categorical variables. This analysis was run to the final sample stratifying by sex, age, Nielsen geographical area, population size, and especially by consumption of standard milk or adapted and fortified milk formulas.

The normality of distribution for each variable was determined using Kolmogorov–Smirnoff. The comparisons between and within groups, and among variables were determined by the Student’s *t*-test, Mann–Whitney U-test, Wilcoxon test, one-way ANOVA test, Kruskal–Wallis test, and/or chi-square test, according to the distribution of the samples and the required outcomes. The significance level was set at *p* < 0.05, with a 95% confidence level. Further statistical models will be run in order to obtain additional outcomes of this study as required.

## 3. Outcome Measures

### 3.1. Primary Outcome Measures

#### 3.1.1. Dietary Habits

Dietary habits were estimated using the quantitative FFQ; the operationalization of the FFQ was done by converting the consumption frequency values into portions and grams of daily and weekly intakes for all food items. These results will be processed using different scores and will indicate the diversity [30,31,32] and variety of food consumption in our study population. 

To evaluate the dietary patterns, we will use different methods, such as principal component analyses (PCA) and cluster analysis, among others [33,34]. For evaluating the overall dietary habits, the consumption frequency of all food items will be categorized as meeting or not meeting the criteria of the dietary guidelines for the Spanish population [35], the food pyramid for the Spanish population [36], and the nutrition goals for the Spanish population [37].

#### 3.1.2. Milk and Dairy Product Consumption

To determine the consumption of dairy products, the frequency of dairy consumption in the last year was asked through the FFQ; additionally, the type and brand of milk consumed was asked using the sociodemographic questionnaire and through the two 24-h DR.

Milk was classified into two groups: cow’s milk and related (e.g., goat) and adapted milk formulas (follow-on milk 2, growth milk 3, and enriched milk).

A detailed extension of the composition of the different infant and adult milk types that currently exist in the market (260 items) was created, obtained from the official manufacturer websites, and added to the software VD-FEN 2.1 [38] for the analysis of the energy and nutrients that these dairy products provide.

#### 3.1.3. Energy, and Macro- and Micronutrient Composition of the Diet

The information collected in the 24-h DR allowed the transformation of food consumption into energy intake, water, macronutrient intake (proteins, fats (total fat, saturated fatty acids (SFAs), monounsaturated fatty acids (MUFAs), polyunsaturated fatty acids (PUFAs), carbohydrates (starch and total sugar), fiber and micronutrient intake (vitamins and minerals).

Mean daily intakes of energy and nutrients were calculated by using the computer software VD-FEN 2.1 [36], mainly based on the food composition tables [39]. Data obtained were evaluated using the dietary reference values of the European Food Safety Authority [40], with the Nutritional Objectives of the Consensus Document of the Spanish Community Nutrition Society [37].

#### 3.1.4. Diet Quality

In order to evaluate the overall diet, it is necessary to analyze the dietary pattern weighted with the different components of a healthy diet. Tools called “diet/dietary quality indexes” evaluate the level of adherence to a specified pattern or a set of recommendations in populations. For the EsNuPI study, the FFQ and the 24-h DR were used to calculate different diet quality indexes, for example, the Kid Med test (Mediterranean diet quality index for children and adolescents) [41,42], among others [43,44,45].

#### 3.1.5. Active and Sedentary Behavior Habits

To estimate the energy expenditure, each activity included in the PABQ was converted using the metabolic equivalents (MET_y_) indicated in the Youth Compendium of Physical Activities [46]. The calculations of energy cost were based on the MET_y_ value from the Youth Compendium, a measured or computed basal metabolic rate (BMR), and the duration of each specific activity, as follows:

Energy cost (kcal) = MET_y_ × BMR (kcal/min) × duration (minutes), where the BMR for boys and girls was predicted using specific Schofield equations [46,47,48].

The physical activity was measured based on the intensity of the activities and was classified according to the latest recommendations of WHO (World Health Organization) guidelines as sedentary (≤1.5 METs), light-intensity activities (1.5–4 METs), moderate-intensity activities (4–7 METs), and vigorous-intensity activities (>7 METs) [49].

The minutes of physical activity per day were also evaluated using the WHO guidelines on physical activity, sedentary behavior, and sleep for children <5 years old [49] on our 1–5-year-old study group. Furthermore, the WHO global recommendations on physical activity for health for 5–17-year-olds [50] were used to evaluate the rest of our study population (five to <10 years old). 

Table 4 shows the recommendations of minutes per day of physical activity by age group according to WHO.

For the number of hours that children spend on screen, the guidelines of the WHO [49] were used to analyze our 1–5-year-old study group and the Ministry of Health, Social Services, and Equality (MSSSI) guidelines [51] were used to analyze the rest of our study population (five to <10 years old). Table 5 shows groups the guidelines of the WHO and MSSSI.

Different institutions include recommendations about children’s hours of sleep like the WHO [50], the National Sleep Foundation (NSF) [52], and the American Academy of Sleep Medicine (AASM) [53] guidelines. Table 6 summarizes the recommendations according to the age group. Our data for the children’s hours of sleep were compared to the NSF recommendations [52].

Finally, to calculate the energy balance, the energy intake obtained in the 24-h DR was compared with the energy expenditure calculated from the basal metabolic rate (BMR) according to Schofield equations [46,47,48] plus the energy derived from physical activity obtained through the PABQ using the Youth Compendium of Physical Activities [46].

### 3.2. Secondary Outcome Measures

#### 3.2.1. Growth and Body Mass Index

Self-reported anthropometric data are valid and recommended for monitoring the prevalence of obesity, particularly for large studies, because of their simplicity and low cost [54].

In the EsNuPI study, the weight and height measurements were reported by the parents or caregivers of the children according to their most recent available pediatrician health book.

The weight and height of the children were evaluated using the WHO international growth patterns based on the analysis of the weight-for-age, height-for-age, weight-for-height, and BMI-for-age indicators. For each child, the *z*-scores of each of these indicators were estimated using the WHO Anthro and WHO Anthro PLUS software (Version 3.2.2, January 2011).

Evaluation of the indicator BMI-for-age was performed at an individual level, and subjects were classified as at risk of overweight, with overweight, or with obesity according to the WHO classification (*z*-score ≥1, risk of overweight; *z*-score ≥2, overweight; *z*-score ≥3, obesity) [55]. The results of this indicator were compared by sex, age groups, Nielsen areas, and type of milk consumed.

#### 3.2.2. Misreporting: Under-Reporting and Over-Reporting of Energy Intake

It is now generally recognized that self-reports of food consumption under-estimate food, energy, food consumption, and nutrient intake. For this reason, the identification of misreports of foods consumed (including under-reporters and over-reporters) is an important aspect in the assessment of uncertainties in food consumption data. Although the 24-h DR and the FFQ used in this study are valid, they could present certain errors because of the under-reporting and over-reporting of food intake.

The plausibility of energy intake was assessed using the cut-off points and the method proposed by Goldberg [56] and updated by Black [57]. BMR was estimated through the Schofield equations [47], considering the age, sex, height, and weight. The specific cut-off points for the age and sex groups were calculated considering the specific reference values and the coefficient of intra-individual variation for energy intake (EI), BMR, and physical activity, according to the method described Nelson et al. [58] and Black [59].

#### 3.2.3. Sociodemographic Information

Multiple factors influence food choice and, thus, diet quality, for example, budget, resources, household structure, and food availability (at a group level), and taste preferences, food attitudes and identity, health motivations, nutritional knowledge, and habitual behavior (at an individual level) [60].

Socioeconomic status was classified based on parental education and occupation. The parental education was classified according to the International Standards Classification of Education [61], which classifies eight levels of education: level (0) early childhood education; level (1) elementary education; level (2) lower secondary education; level (3) upper secondary education; level (4) post-secondary non-tertiary education; levels (5)–(8) tertiary education (level 5, short stage of tertiary education; level 6, tertiary education degree; level 7, masters and specialization level; level 8, doctorate level).

The occupation was classified according to National Classification of Occupations of Spain [62]: (1) directors and managers; (2) technical and professional researchers and intellectuals; (3) technician; (4) accounts clerk, administrative work, and office employees; (5) restaurant and catering work, salespeople, etc.; (6) agricultural producers, stockbreeders, fish producers, and forestry employees; (7) craftspeople, people working in manufacturing and construction industries; (8) installation and machinery operators, and assemblers; (9) elementary occupations; (10) military occupations.

## 4. Discussion 

The multi-approach proposed methodologies described in this paper aim for the first time to evaluate the food habits, dietary patterns, and nutrient intake, as well as physical activity and sedentary behaviors, of Spanish children (one to <10 years). The protocol was also focused on splitting the population according to dairy product consumption, specifically in terms of types of milk and their role in the dietary pattern and quality of the overall diet.

In the EsNuPI study, the use of dietary surveys (FFQ and 24-h DR) was considered because of their reliability and usefulness to evaluate the dietary habits, dietary patterns and energy, food consumption, and nutrient intake of the studied population [22,23].

The research team took advantage of the opportunity to collect data using a personal FFQ, 24-h DR, and telephone 24-h DR, as well as the PABQ questionnaire; all the information was collected by trained interviewers in order to complete the information about each child’s lifestyle. The data obtained will enable running specific comparisons and associations in order to respond to the goals of the study. 

There were national and international studies that investigated children’s lifestyles including diet, dietary patterns, physical activity, and sedentary behaviors [7,8,63,64]. However, there is currently no current information on children (one to <10 years) in Mediterranean populations. In Spain, the ALSALMA study was useful in terms of the description of food intake, but several limitations can be identified, especially the absence of an FFQ to determine the habits of infants and toddlers (0–36 months of age). The study did not attempt to perform any correlation between diet and socioeconomic status or educational status of the parents. Physical activity data were not collected; therefore, it was not possible for the researchers to estimate the energy balance and under- and over-reporting of energy intake [7].

The ENALIA study was designed to collect dietary intake and information of dietary habits in Spanish children and adolescents. This study only collected the physical activity of children ≥10 years old, and it was carried out during an economic crisis, which is known to be an important factor for the dietary intake of families [8,65].

Furthermore, international studies were conducted. The United Kingdom (UK) National Diet and Nutrition Survey (NDNS) Rolling Program (RP) (2008–2014) is a continuous cross-sectional survey, designed to assess the diet, dietary intake, and nutritional status of a representative sample from the general population aged 18 months and upward. The NDNS that was conducted in 2012 comprised a four-day diary and an evaluation of the physical activity levels of children aged three to 15 years. However, in the group aged 1–3 years, the physical activity was not collected [63].

The Feeding Infants and Toddlers Study (FITS, 2016) and the Kids Nutrition and Health Study (KNHS, 2009–2012) were large-scale cross-sectional surveys designed to explore dietary patterns, nutrient intake, and food sources of nutrients among infants and children (0–13 years old) in different countries of the world. In each FITS and KNHS participant country, dietary intake was assessed using 24-h DR on one, two, or more days. The age groups and food grouping systems were not consistent, the level of detail varied between countries, and not all country surveys recorded details about the meals or times when foods were consumed [64].

Finally, the IDEFICS and I.Family studies shared objectives and outcomes with our study, and they will be helpful to discuss our future publications. Even though this was a project designed at an international level, it did not include a representative sample of the Spanish population. Hence, it will be interesting to compare outcomes of both studies [10,11,12].

We consider that the EsNuPI study will provide valuable and new information about dietary habits, sociodemographic data, and physical activity and sedentary behaviors for a wider age range of the Spanish pediatric population (one to <10 years) in the current economic situation, using different tools and methods of collection.

Additionally, and as already mentioned, different valuable dietary surveys were conducted in Spain, although, to the best of our knowledge, none attempted to determine the role of adapted and fortified milk formula consumption in this population. This food group has special relevance in children’s diets (especially in early childhood); thus, the consumption of dairy products and the specific role of adapted and fortified milk formulas will be analyzed in this study.

The methods we followed to complete this study show a series of strengths that allowed a good quality of the information obtained. In this study, the data collection method was not invasive; consequently, the interviews were carried out with the children’s parents or caregivers. This fact is also an advantage due to the ease for parents to remember established routines and mealtimes. For this reason, we expected less bias in their answers [66]. Another strength would be the quality of the survey tools that were specifically adapted to this population and that were verified and modified during the pilot study. We used the computer application CAPI for the collection of all the data to make this process easier and more efficient, and the VD-FEN 2.1 software [38] allowed the conversion of all the information into a database that can be used in different statistical programs. Finally, the use of supporting material such as the “tables of common home measures and habitual portion sizes for Spain” [26] and the “photo guide of common portions sizes of Spanish foods” [27], built using the “pilot study for the assessment of nutrient intake and food consumption among kids in Europe” (PANCAKE) standards [67] to improve the quality of the answers obtained during the interviews, is another recognized strength of the present study.

We recognize a series of limitations in this study; for example, even though the interviewers were previously trained by experienced dieticians/nutritionists, they were professionals of different areas and degrees (e.g., law, history, nursing, and designer degrees, administrative work, etc.).

Moreover, the anthropometric data declared by parents based on the child´s health card may introduce somewhat misreporting responses. Also, potential biases may occur when self-reported data are analyzed, as is the case for dietary recall questionnaires. In epidemiologic studies of dietary factors as determinants of diseases, FFQs are commonly used to assess long-term or usual food consumption [68]. FFQs are based on an individual’s recall of usual intake over a specified time and, thus, are subject to measurement error, which leads to an under- or over-estimation of food consumption.

However, two 24-h DRs were performed to control and reduce the measurement error and to evaluate the validity of the food frequency questionnaire. Furthermore, the instruments that evaluated the dietary quality were previously validated and adapted to our studied population in order to reflect the reality of their diet [69]. Additionally, misreporting was determined in our study, and secondary analyses will be run with plausible data [70]. 

Another limitation was the use of a subjective method for measuring physical activity. The use of accelerometers combined with self-reported data could probably provide more accurate information; in our case, the use of accelerometers was not possible due to the ages of the youngest group in our study population. Furthermore, another limitation was the use of the Youth Compendium of Physical Activities for children under six years old, due to the lack of information for activities under this age and the lack of MET values for this age range. Finally, although the sample is representative of Spain, we would have liked to be able to enroll more participants, which would enlarge our sample size and, consequently, would reduce the sampling error.

## 5. Conclusions 

The EsNuPI study is the first study targeted at the one- to <10-year-old age group to collect data about dietary habits, dietary patterns, physical activity, and sedentary behaviors in the Spanish population. Considering that the protocol was designed based on the best available evidence and previous experience, the EsNuPI study will contribute to provide valuable information for the development of dietary guidelines, and nutritional and health policies focused on improving the health of the child population.

## Figures and Tables

**Table 1:** Inclusion and exclusion criteria for the EsNuPI study.

Inclusion Criteria	Exclusion Criteria
Healthy participants between 1 and <10 years old without diseases entailing changes in their dietary patterns.	Children with any disease that causes changes in their dietary patterns or any dietary restriction, or children following a therapeutic diet due to recent surgery or any other medical prescription and pathology.
Children whose parents/caregivers report that they are following their habitual diet.	Children whose parents reported that they were not following a normal diet for any reason, including a transitory pathology (cold, flu, gastroenteritis, chickenpox, etc.) at the time of the fieldwork.
Children whose parents or caregivers can read and understand the questionnaires properly.	Children following a vegan diet.
	Children who live in an institution (boarding schools, nursing homes, hospitals, host institutions, etc.).
	Children related to Madison MK or Lactalis employees *.
	Children whose parents or caregivers did not provide a signed consent form for their participation or did not complete any study phase.

* Children related to employees of Madison MK, company in charge of the interviews, and Lactalis, the flagship company of “Instituto Puleva de Nutrición”, the financing collaborator of this project are excluded in order to avoid bias because the parents could be aware of the study and their answers could be conditioned, and bias due to the consumption of Lactalis-branded product within these families.

**Table 2:** Distribution of the sample for the EsNuPI study.

		Sample (*n*)				
		Targeted Sample (*n* = 1500)		Final Sample (*n* = 1514)		
		Reference *	Booster **	Reference *	Booster **	Total
		*n* = 750	*n* = 750	*n* = 742	*n* = 772	*n* = 1514
Sex	Boys	375	375	374	385	759
	Girls	375	375	368	387	755
Age (years)	1 to <3	250	250	171	303	474
	3 to <6	250	250	257	276	533
	6 to <10	250	250	314	193	507

* Sample of non-vegan one- to <10-year-old children representative of the Spanish population living in urban areas. ** Booster sample of non-vegan one- to <10-year-old children who consumed adapted and fortified milk formulas and lived in urban areas.

**Table 3:** Main food groups and subgroups and reference portion sizes used in the EsNuPI study quantitative food frequency questionnaire.

Food Groups	
**Dairy products** Breast milk (150 mL), infant formula, toddler’s milk formula, cow’s milk, enriched milk, and specially formulated milk, 200 mL Vegetable drinks (200 mL) Yogurt (125 g) Fresh cheese (15–30 g) and cured cheese (15 g) Sugared dairy dessert (125 g) and ice cream (120 g) Other dairy products (condensed milk, 10 g; cream, 20 g; and flavored milk, 200 mL)	**Oils and fats** Olive oil (10 g) Other vegetable oils and lard (10 g) Margarine and butter (12 g)
**Eggs, meat, and fish** Eggs (64 g) Chicken and turkey with or without skin, medium-fat meat, fatty pork, lamb meat, rabbit meat, or liver and other viscera (55 g) “Serrano” ham, cooked ham, sausages, other processed meats (15 g) Fish: white fish (55 g), bluefish (small and large, 55 g), fresh seafood (90–100 g), and canned fish (25 g)	**Bakery products** Cookies and cakes (25–50 g) Homemade bakery products (50 g) Sugary cocoa (5 g), nougat (20 g), and shortbread cookies (45 g)
**Vegetables and potatoes** All kind of vegetables (55 g) Potatoes (55–80 g)	**Miscellaneous** Industrial pre-cooked products (25–50 g) Homemade pre-cooked products (25–50 g) Sauces (8 g) Condiments (0.25 g) Sugar, honey, and marmalade (10 g) Low- or no-calorie sweetener (0.05 g) Snacks (fried snacks, 50 g)
**Fruits and nuts** All kinds of fresh fruits (100–200 g) Olives (20 g) Fruits with juice and fruits with syrup (50 g) Dried fruits (20 g)Nuts (17.5 g)	**Beverages** Sugar-sweetened carbonated beverages or low-calorie carbonated beverages (200 mL) Natural juices and commercial juices (200 mL) Mixtures of fruit juice and milk, sugar-sweetened or low-calorie sweetened (200 mL)
**Legumes, cereals, and pasta** Legumes (40 g) Bread (20–25 g) Breakfast cereals (30 g) Rice and pasta (40 g)	**Homemade and commercial baby food, homemade and commercial baby porridge** Homemade baby food (200 mL) Fruit commercial baby food (200 mL) Vegetable baby food (200 mL) Vegetable commercial baby food (200 mL) Vegetables with meat and/or fish homemade baby food (200 mL) Cereals with milk homemade baby porridge (200 mL) Cereals with milk commercial baby porridge (200 mL)

Gil-Campos, M. Personal communication from Mercedes Gil-Campos, as coordinator of the Meli-Pop Project (Reference PI18/0093 del Fondo de Investigaciones Sanitarias, Instituto Carlos III), 2018, modified.

**Table 4:** World Health Organization guidelines on physical activity behavior.

AGE GROUP (years)				
	<1	1–2	3–4	5–17
World Health Organization [49,50]	At least 30 min every day	At least 180 min every day	At least 180 min every day, with 60 min of moderate-to-vigorous intensity	At least 60 min every day of moderate-to-vigorous intensity

**Table 5:** Screen time recommendations by age group according to the World Health Organization and the Ministry of Health, Social Services, and Equality.

	AGE GROUP (years)			
	<1	1–2	3–4	5–17
World Health Organization [49,50]	0 min	1 year: 0 min 2 years: no more than 60 min	No more than 60 min	-
Ministry of Health, Social Services and Equality [51]	**0 to <2**		**2–4**	**5–17**
	0 min		No more than 60 min	No more than 120 min

**Table 6:** Sleep time recommendations by age group according to the World Health Organization, National Sleep Foundation, and American Academy of Sleep Medicine.

	AGE GROUP (years)			
	<1	1–2	3–4	5–17
World Health Organization [50]	0–3 months 14–17 h 4–11 months 12–16 h	11–14 h	10–13 h	-
National Sleep Foundation [52]	**<1**	**1–2**	**3–5**	**6–13**
	0–3 months 14–17 h 4–11 months 12–15 h	11–14 h	10–13 h	9–11 h
The American Academy of Sleep Medicine [53]	**<1**	**1–2**	**3–5**	**6–12**
	4–12 months 12–16 h	11–14 h	10–13 h	9–12 h
